# Case Report: Improvement in neurologic symptoms and remission following non-operative management of locally advanced cutaneous squamous cell carcinoma of the head and neck following cemiplimab therapy: two cases

**DOI:** 10.3389/fonc.2026.1975694

**Published:** 2026-10-15

**Authors:** Elijah Lee, Wolfram Samlowski, Brandon Reynolds, Kaviyon Sadrolashrafi

**Affiliations:** 1 Kirk Kerkorian School of Medicine at University of Nevada (Las Vegas UNLV), Las Vegas, NV, United States; 2 Nevada Oncology Specialists, Las Vegas, NV, United States; 3 Reynolds Plastic Surgery, Las Vegas, NV, United States; 4 Division of Dermatology, Department of Medicine, David Geffen School of Medicine at University of California, Los Angeles, Los Angeles, CA, United States

**Keywords:** checkpoint inhibitor, keratinocyte carcinoma, neoadjuvant therapy, perineural extension, skin cancer

## Abstract

**Background:**

Cutaneous squamous cell carcinoma is a common skin malignancy in aging populations. Surgical resection and radiotherapy can result in significant morbidity, as well as functional and cosmetic impairments. PD-1 antibodies are promising treatments for locally advanced disease.

**Case presentation:**

Two elderly men (aged 71 and 73) with locoregionally advanced head and neck tumors received neoadjuvant treatment with cemiplimab (350 mg IV every 3 weeks). Both patients presented with neurologic symptoms concerning for nerve involvement; however, perineural spread could not be identified radiographically or by limited biopsies. Both patients achieved a significant clinical response after only four doses of cemiplimab. After 4 additional doses, both patients underwent elective treatment discontinuation and surgery and radiotherapy were omitted. With over 12-month follow-up, both patients remained free of recurrence. Treatment was well-tolerated with only Grade 1 pruritus.

**Conclusion:**

These observations are promising and suggest the feasibility of response-adapted management following cemiplimab therapy in selected patients with advanced squamous cell skin cancer with resolution of neurologic symptoms. This approach may allow for omission of surgery and radiotherapy in selected patients. Further investigation to define appropriate candidates for this approach appears warranted.

## 1 Introduction

Cutaneous squamous cell carcinoma (CSCC) represents the second most common skin cancer worldwide, accounting for approximately 20% of all keratinocyte-derived malignancies (1). It is estimated that more than 1 million cases of CSCC occur annually in the United States. The precise incidence of keratinocyte carcinomas is difficult to determine because these cancers are not reportable to U.S. national cancer registries, including the Surveillance, Epidemiology, and End Results Program (SEER) registry (2).

The CSCC mortality rate among individuals aged 65 or older increases progressively with age, rising from 2.5 per 100, 000 population in those aged 65–69 to 21.28 per 100, 000 in those aged 95 years or older. Projections indicate that the number of new cases and deaths attributable to non-melanoma skin cancer will increase by approximately 1.5-fold between 2020 and 2044 (3). Contributing factors to this rising incidence include the increasing average age of the population, greater cumulative sun exposure, and a growing prevalence of immunosuppressive conditions (4).

Although surgery or radiotherapy remain standard treatment for most patients with small primary CSCC, a minority of patients present with locoregionally advanced disease, representing approximately 2-5% of all cases (5, 6). Surgical management of these higher-risk CSCC usually requires more extensive resections followed by adjuvant radiotherapy. This can lead to significant treatment-related complications, as well as cosmetic and functional impairment (7), particularly in CSCC tumors arising in the head and neck region, which make up about 80% of the advanced cases (8).

Recent progress in immunotherapy has reshaped management options for locally advanced or unresectable CSCC. Agents targeting the programmed death-1 (PD-1) pathway, such as cemiplimab, nivolumab and pembrolizumab, have demonstrated high clinical response rates (9–12). These responses have frequently proven durable, with longer-term follow-up (13, 14). These results have led investigators to explore neoadjuvant PD-1 blockade, using immunotherapy prior to surgery, to induce radiologic or pathologic responses to shrink tumors and improve resectability (8, 15). Most of these trials have incorporated planned surgery and radiotherapy. The feasibility of non-operative therapy of locally advanced CSCC is starting to be explored (16). Several recent studies have begun to examine definitive non-operative cemiplimab treatment with response-adapted reductions in surgical resection and radiotherapy (17–19).

We report two cases of non-operative management of elderly male patients with large, neglected CSCC tumors treated with neoadjuvant cemiplimab (350 mg IV every three weeks). Both patients had repeatedly declined surgery and radiotherapy. These patients exhibited a dramatic response following cemiplimab therapy with improvement in peripheral nerve symptoms. Both patients achieved a complete remission.

## 2 Case reports

### 2.1 Case 1

A 71-year-old man with a history of anxiety and depression presented with a 4 cm, ulcerated, well-differentiated squamous cell carcinoma located on the left cheek (**Figure 1A**). The lesion had been present for three years, with recent enlargement, recurrent hemorrhage, and functional peripheral nerve impairment. He had developed the inability to fully close the left eye, with a left facial droop for several months. Despite a significant history of early life sunburns, the patient has no prior history of cutaneous malignancy.

**Figure 1:**
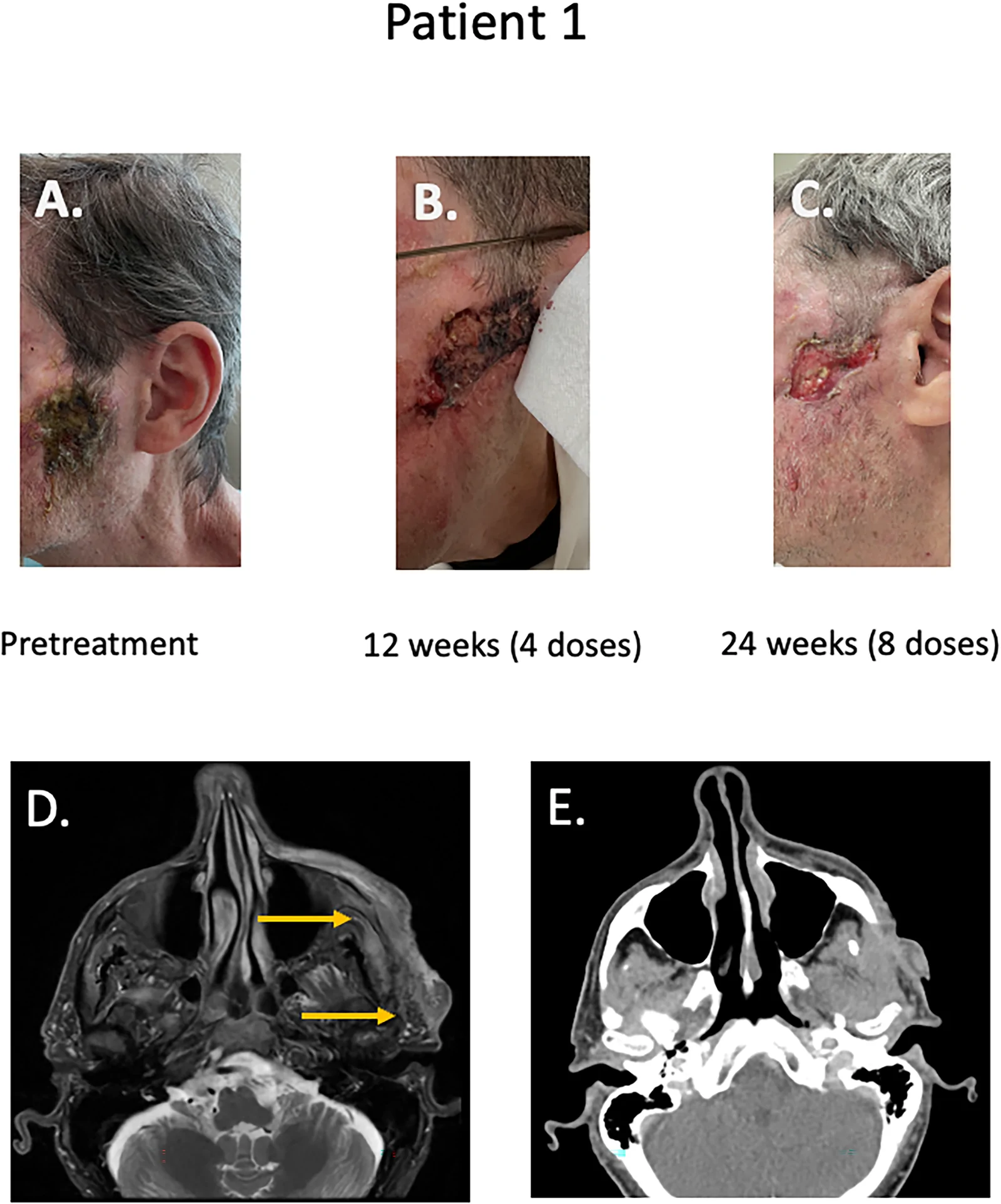
Clinical photographs documenting the pretreatment neoplasm in patient 1 and the time course of clinical response during cemiplimab therapy [Panel **(A)**, pretreatment; Panel **(B)**, after 4 weeks of treatment, Panel **(C)**, after 8 weeks of treatment]. Panel **(D)**, CT image of patient 1 pretreatment, Panel **(E)**, CT image of patient 1 at 4 months following treatment initiation.

Due to the size of the lesion and left facial weakness, there were concerns about potential deep tissue invasion and involvement of cranial nerve VII. Radiologic imaging revealed a large, deeply invasive preauricular tumor (AJCC stage T3, N0, M0) (**Figure 1B**). Perineural extension could not be visualized via radiologic imaging or limited biopsies. The tumor’s size, deep tissue invasion, and associated functional impairment contributed to its characterization as regionally advanced. After multidisciplinary discussion, the patient was treated with neoadjuvant cemiplimab therapy (350 mg i.v. every three weeks), with the goal of cytoreduction, to be followed by potential resection. Following four cycles, regression of the skin lesion was observed (**Figure 1B**). This treatment also resulted in significant improvement in the patient’s left facial weakness. An additional four cycles of consolidation therapy were administered with further improvement (**Figure 1C**). Cemiplimab therapy was well tolerated, with no reported acute or delayed adverse effects. Radiographs revealed significant reduction in the preauricular tumor (**Figure 1E**). Comparison of pre and post-treatment biopsies of the primary site confirmed a pathologic complete response (pCR) (**Figure 2**). Planned surgical wide excision and radiotherapy were deferred. The patient currently remains in remission more than 12 months following the start of therapy. The treatment strategy schema is shown (**Figure 3**).

**Figure 2:**
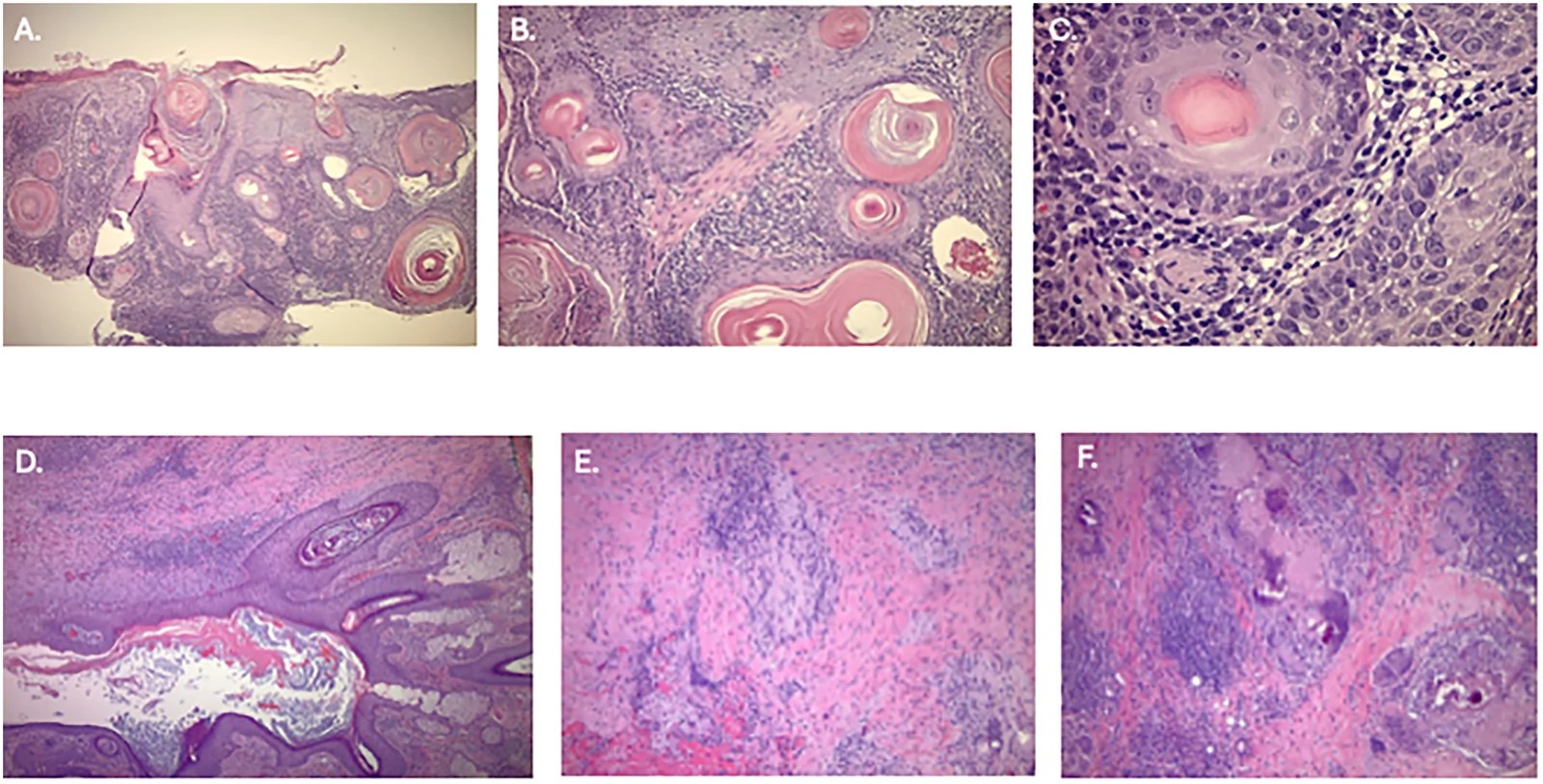
Pre-treatment biopsy in patient 1: Proliferation of atypical squamous cells showing downward growth from overlying epidermis, associated with abundant keratinization, mild pleomorphism, and mitotic figures (blue arrows). [Panel **(A)**, hematoxylin and eosin stain 40x magnification, Panel **(B)**, H&E stain 100x magnification, Panel **(C)**, H&E stain 200x magnification]. Post-treatment biopsy: No residual SCC with marked scarring fibrosis, chronic and granulomatous inflammation, and calcification. [Panel **(D)**, H&E stain 40x magnification, Panel **(E)**, H&E stain 100x magnification, Panel **(F)**, H&E 100x magnification].

**Figure 3:**
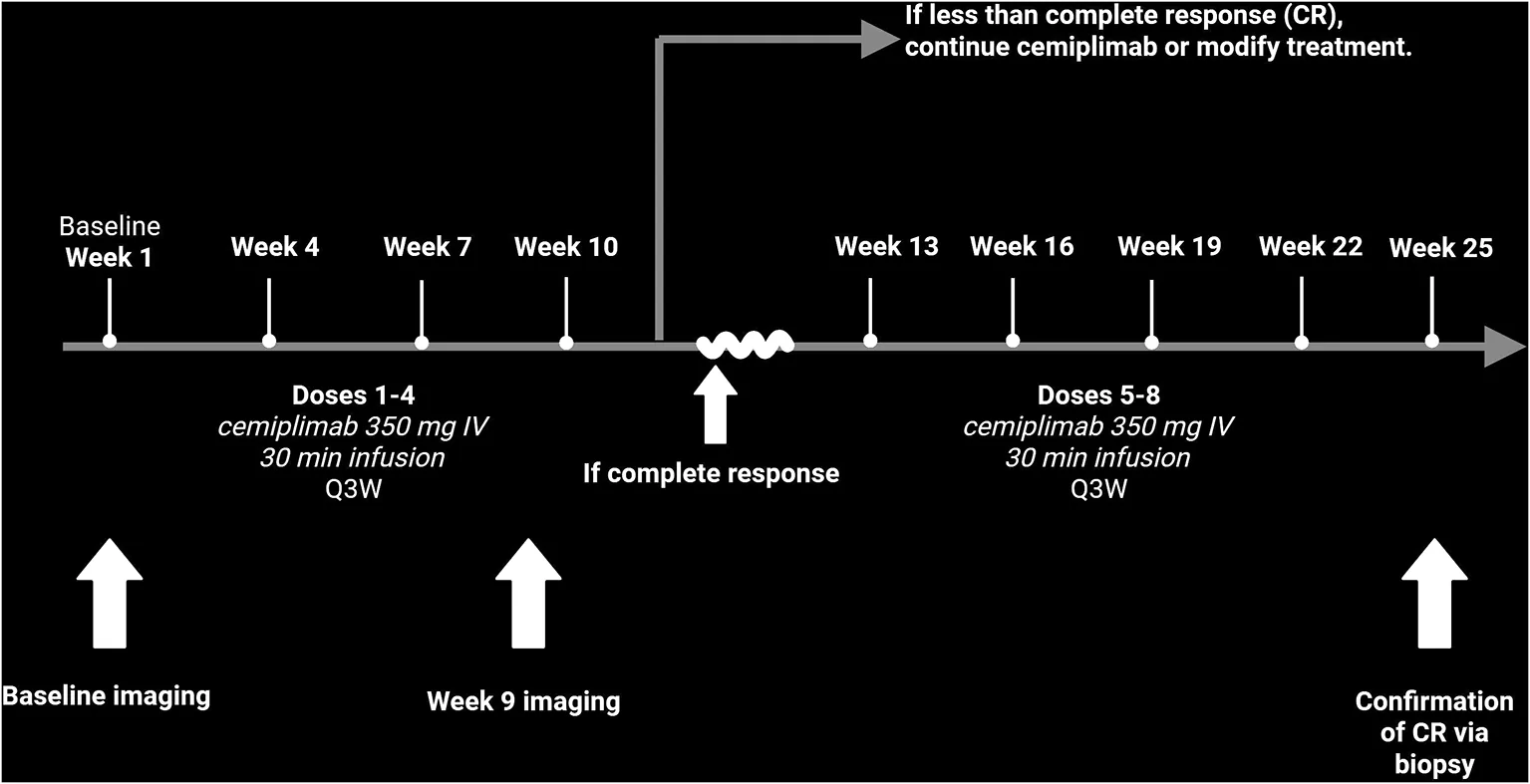
Cemiplimab treatment schema.

### 2.2 Case 2

A 73-year-old man with a history of hypertension and osteoarthritis presented with a large 4 x 6 cm invasive squamous cell carcinoma of the right frontal scalp. The lesion had been present for approximately seven years (**Figure 4A**). The patient had repeatedly declined surgery and radiotherapy. He sought dermatologic evaluation following the onset of bleeding from the lesion. He also had pain and numbness of the lower forehead.

**Figure 4:**
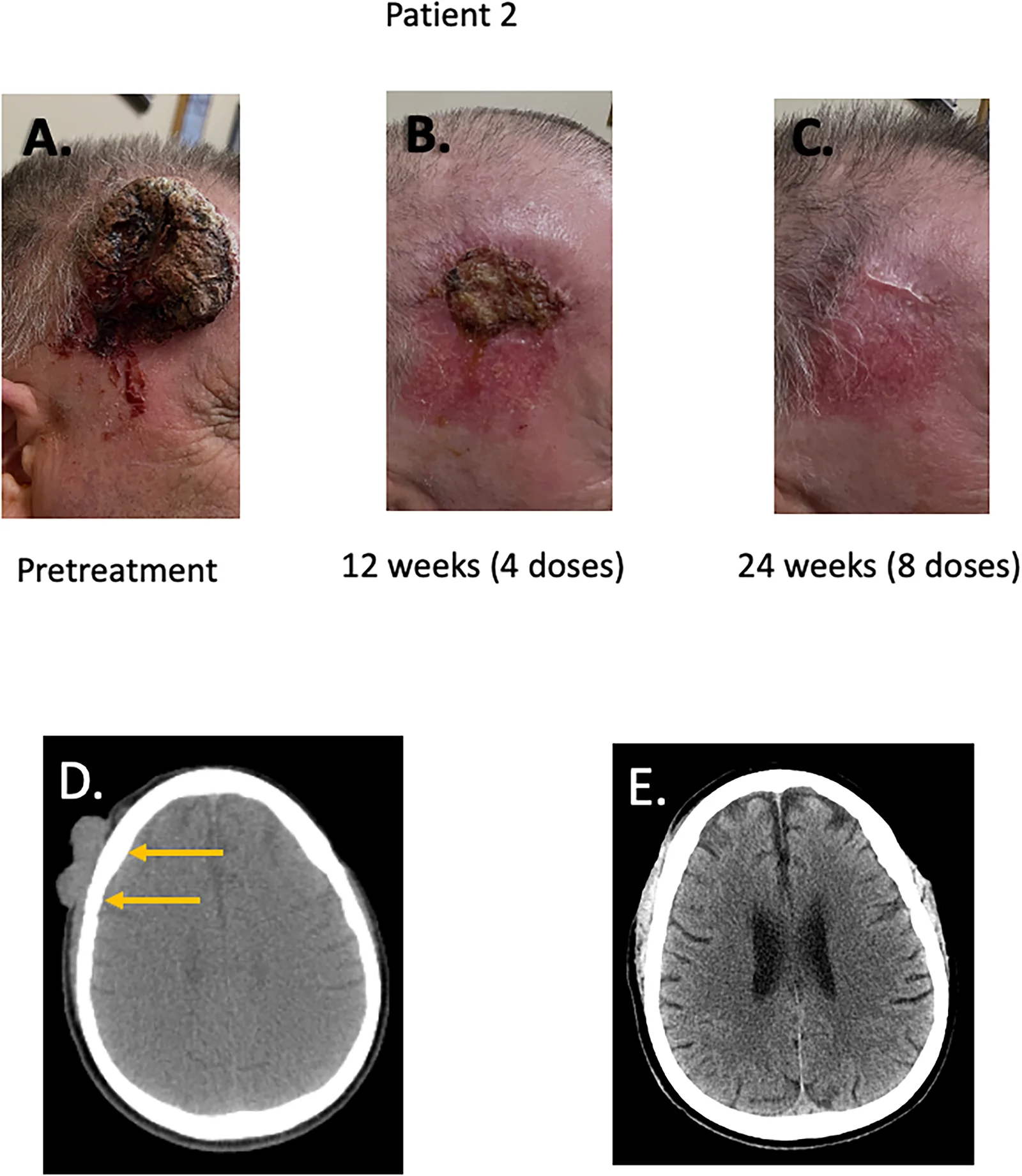
Clinical photographs documenting the pretreatment neoplasm in patient 2 and the time course of clinical response during cemiplimab therapy [Panel **(A)**, pretreatment; Panel **(B)**, after 4 weeks of treatment, Panel **(C)**, after 8 weeks of treatment]. Panel **(D)**, CT image of patient 2 pretreatment (21 June 2024). Panel **(E)**, CT image of patient 2 at 5 months following treatment start (4 November 2024).

A biopsy performed on May 16, 2024, diagnosed a well-differentiated squamous carcinoma. As part of the diagnostic workup, a CT scan of the face and neck was performed. The imaging revealed the primary scalp mass (AJCC stage T3, N1, M0) (**Figure 4D**). The patient had numbness of forehead and right eyebrow area. However, perineural tumor extension was not visualizable on scans. Radiographs also identified an asymmetrically enlarged right parotid and posterior jugular lymph nodes measuring up to 1cm (not shown). Ultrasound-guided cytologic sampling of the parotid confirmed a tumor metastasis.

Following multidisciplinary review, the patient was treated with neoadjuvant cemiplimab (350 mg IV every three weeks), with the goal of cytoreduction and potential delayed resection. After four cycles, complete tumor regression was noted, along with resolution of paresthesia (**Figure 4C**). Following four consolidation cycles, the patient maintained an apparent clinical (**Figure 4D**) and radiographic complete remission (**Figure 4E**) with complete healing of the skin at the site of prior tumor ulceration. The patient declined post-treatment biopsies and imaging of the previous tumor site to confirm the apparent remission. The only toxicity was mild pruritus of the bilateral forearms and posterior scalp. The patient died of unrelated infection 1.5 years following completion of treatment, while in remission.

## 3 Discussion

CSCC is one of the most common malignancies in the United States, with an estimated incidence of more than 1 million new cases per year (2). This rising burden reflects an aging population, greater ultraviolet light exposure, predisposing low-grade lymphomas, and expanding use of immunosuppressive therapies. Locally advanced CSCC represents a small but clinically significant subset of cases, estimated to comprise 2-5% of all CSCC, and is associated with higher risks of recurrence and distant dissemination (5, 6). The precise frequency of locally advanced or metastatic CSCC in the United States is unknown, as this is not a reportable cancer. It has been estimated that 30, 000 patients a year may be affected (1, 5).

Management of these large, neglected tumors is challenging, especially when lesions arise near cosmetically and functionally critical regions in head and neck sites. Traditionally, surgery and radiotherapy were the main treatment modalities used to treat locoregionally advanced CSCC. It should also be noted that complete resection of tumor in patients with peripheral nerve involvement would likely induce more dense and permanent loss of nerve function. As most CSCC patients are elderly, with significant co-morbidities, chemotherapy has not been widely utilized as a treatment option. More recently, treatment of locally advanced or metastatic CSCC with checkpoint inhibitor antibodies directed against PD-1 or PD-L1 have has demonstrated a high rate of clinical response and durable remissions (14, 20–23).

Clinical trials have attempted to take advantage of promising complete response rates following neoadjuvant immunotherapy for treatment of potentially resectable CSCC (24, 25). In a phase II multicenter, nonrandomized trial, Gross et al. evaluated neoadjuvant cemiplimab therapy in 79 patients with resectable stage II-IV (M0) CSCC. When patients underwent planned surgical resection (after 2 cycles), there was a pathologic complete response rate (pCR) of 51% and a major pathologic response rate (MPR: ≤ 10% viable tumor) of 13% (26). Another case series has also supported this approach (27).

Non-operative management following PD-1 blockade has already been described in larger studies. Kavanagh et al. reported an estimated two-year disease-specific survival of 90% (95% CI, 80%–100%) and progression-free probability of 82% (95% CI, 72%–94%) among 51 patients treated with cemiplimab alone (17). Yaniv et al. examined the potential for neoadjuvant cemiplimab to reduce the extent of surgery by comparing response-adapted oncologic surgery against standard resection (18). The DISCERN trial highlighted how radiologic and pathologic responses may significantly differ (19). Together, these studies provide context for response-adapted management.

More recently, Ladwa et al. evaluated a treatment de-escalation strategy following neoadjuvant therapy with pembrolizumab (16). This phase II, multicenter nonrandomized study enrolled 27 patients with resectable stage II-IV (M0) CSCC. 67% of patients were in stage IV, and 89% had masses localized to the head and neck. Patients received neoadjuvant pembrolizumab (200mg every three weeks for four cycles) followed by “response-adapted” management. After four doses of pembrolizumab, 63% of patients achieved a clinical or radiologic complete remission. Surgery and radiotherapy were then omitted in 48% of patients. There were no tumor recurrences among patients who achieved a complete response (16). Patients with less than a complete response underwent subsequent surgery and radiotherapy. With a median follow-up of 18 months, overall event-free survival was 74%.

These emerging approaches should also be considered within contemporary treatment guidelines. The 2026 European interdisciplinary guideline continues to recommend surgery as first-line treatment for common primary CSCC and anti-PD-1 therapy as first-line system treatment when curative surgery or radiotherapy is not appropriate. These recommendations do not establish routine omission of definitive local therapy after an apparent response to immunotherapy (28).

It is important to note however, that our findings should be considered alongside other recent evidence regarding cemiplimab therapy in older adults, as well as evolving approaches to advanced CSCC management (24). In a retrospective cohort of 106 patients, Kseienski et al. reported similar response rates across age groups, consistent with the clinical benefit observed in our two patients. However, grade ≥3 immune-related adverse events occurred in 27.4% of overall patients, highlighting important tolerability considerations (29). Higher pretreatment neutrophil to lymphocyte ratio was also associated with shorter survival, suggesting its potential value as a prognostic biomarker (29). Nevertheless, Kreft et al. highlight that deep responses to neoadjuvant PD-1 blockage may allow less extensive local treatment and, in selected patients, omission of surgery altogether (30).

An ongoing clinical question is how long therapy should be continued once patients achieve complete remission. We have previously examined this question in a cohort of 21 patients with locally advanced or metastatic CSCC treated with cemiplimab (31). In this series of 21 patients, 15 (71.4%) achieved a clinical or radiographic complete remission (CR). After confirmation of a complete remission with scans or biopsies, all 15 patients underwent elective treatment discontinuation. Patients who achieved a CR received only a median of 8.0 ± 3.6 doses of cemiplimab. The median treatment duration was only 5.3 ± 3.7 months. With a median follow-up of four years, 14/15 (93.3%) patients have remained in a durable complete remission. These findings further support the durability of complete remissions in advanced CSCC, allowing early treatment discontinuation (31). It should be noted that other authors have shown varying results of early treatment discontinuation (32, 33). Our results and those of Ladwa et al. emphasize the importance of achieving a complete remission prior to consideration of elective treatment discontinuation (32).

Gili et al. provide additional context for response-adapted treatment de-escalation in CSCC. Their systematic review and meta-analysis included seven prospective studies comprising 270 patients. Among the five studies reporting pathological complete response (193 patients), the pooled rate was 45.7% (95% CI, 37.3-53.4%) (34). Similarly, Boutros et al. examined early cemiplimab discontinuation in a retrospective cohort of 48 patients with advanced CSCC. Nineteen patients discontinued treatment without disease progression. Three were due to adverse effects while sixteen were because of patient or physician preference after achieving a partial response, stable disease, or a complete response. No disease relapse was reported during a median follow-up of 9.2 months after discontinuation (33).

These findings demonstrate that neoadjuvant immune checkpoint inhibition can produce significant tumor responses, supporting investigation of reduced treatment intensity in complete and near-complete responders. However, pathological response rates and favorable outcomes following neoadjuvant therapy have not yet established the likelihood of maintaining long-term disease control, particularly after early discontinuation of cemiplimab, especially when radiotherapy and surgery are also omitted.

Our cases extend these observations by describing neurologic improvement in two patients with loco-regionally advanced CSCC which would have been unlikely following aggressive surgical resection, This included persistent complete remission for at least 12 month follow-up after a short course of cemiplimab. Follow-up measured from treatment initiation should be distinguished from the post-discontinuation interval, as reported by Boutros et al. (33). Along with prior publications, this report confirms substantial clinical and pathological responses following anti-PD-1 directed immunotherapy, providing further rational for investigating where selected patients can undergo treatment de-escalation. Response adapted studies such as De-Squamate offer preliminary support for surgical omission in patients with rigorously confirmed responses (16).

Retrospective discontinuation studies suggest that remissions may persist after elective discontinuation of immunotherapy, especially in patients who achieve a confirmed complete remission (16, 31, 33, 34). However, while these findings are intriguing, they have not yet established that an abbreviated course of cemiplimab can routinely replace surgery or radiotherapy. Our two cases support further investigation of response-adapted treatment. Critical unanswered questions include how to exclude residual primary and nodal disease, which patients can safely avoid local therapy, and how long immunotherapy should continue after apparent remission. Longer follow-up, prospective studies, and randomized control trials assessing outcomes after discontinuation are critical in answering these questions and for determining the likelihood of sustained disease control.

## 4 Conclusions

These two cases illustrate the potential feasibility of response-adapted non-operative management with complete tumor regression and clinical resolution of peripheral nerve symptoms following cemiplimab treatment in selected patients with advanced head and neck cSCC. However, given the small number of patients in this case report with relatively short follow-up, our results should be considered hypothesis-generating. The resolution of peripheral nerve symptoms is particularly intriguing. Longer follow-up and prospective studies are needed to define appropriate patient selection, response assessment, and treatment duration. Any consideration of omitting definitive local therapy should involve multidisciplinary review, informed patient participation, and careful ongoing surveillance. The role and timing of surgery and radiotherapy in locally advanced CSCC may need to be reconsidered in patients who achieve a complete remission following immunotherapy.

## Data Availability

The data analyzed in this study is subject to the following licenses/restrictions: Data restricted by US HIPAA regulations, de-identified patient information provided upon request to corresponding author. Requests to access these datasets should be directed to wsamlowski1@gmail.com.
